# Preliminary In Vitro Screening of Structure-Dependent β-Hydroxybutyrate Responses to Dietary Fatty Acids in Hepatocyte Models

**DOI:** 10.3390/nu18122021

**Published:** 2026-06-21

**Authors:** Xiaojing Liu, Fei Pan, Yandan Wang, Wei Wei, Jun Jin, Xingguo Wang, Zhe Cui

**Affiliations:** 1 State Key Laboratory of Food Science and Resources, School of Food Science and Technology, Jiangnan University, Wuxi 214122, China; xiaojingliu_kmust@163.com (X.L.); yandanwang1013@163.com (Y.W.); weiw@jiangnan.edu.cn (W.W.); junjin@jiangnan.edu.cn (J.J.); 2 Food Laboratory of Zhongyuan, Luohe 462300, China; 3 State Key Laboratory of Resource Insects, Institute of Apicultural Research, Chinese Academy of Agricultural Sciences, Beijing 100093, China; yunitcon@yeah.net; 4 School of Life Sciences, Anhui Normal University, Wuhu 241000, China; 5 College of Sports Medicine and Rehabilitation, Shandong First Medical University & Shandong Academy of Medical Sciences, Tai’an 271016, China

**Keywords:** fatty acids, β-hydroxybutyrate, hepatocyte models, structure–response relationship, machine learning, in vitro screening

## Abstract

Background/Objectives: Controlled comparisons of extracellular β-hydroxybutyrate (β-HB) responses induced by individual dietary fatty acids (FAs) remain limited. This study established a preliminary hepatocyte-derived in vitro assay for comparing FA-associated β-HB responses and used exploratory descriptor-based modeling for hypothesis-generating FA ranking. Methods: Dose- and time-dependent extracellular β-HB accumulation induced by nineteen dietary FAs was quantified in HepG2 and AML12 cells under nutrient-deprived assay conditions. For exploratory descriptor-based modeling, the 19-FA HepG2 dataset was split into 15 training compounds and four held-out compounds. Model stability was further assessed using repeated random splits, LOOCV, permutation testing, MCFA sensitivity analyses, and simple structural baseline models. Four additional structurally related FAs were tested only for preliminary experimental ranking assessment, not as an independent external test set. Results: C8:0 and C10:0 consistently induced the highest extracellular β-HB accumulation, whereas most long-chain saturated FAs and very-long-chain monounsaturated FAs showed lower responses. The single four-compound held-out subset yielded an apparent R^2^ of 0.875, but repeated random-split assessment showed substantial split-dependent variability, and simple baseline models performed similarly to GradientBoosting. Conclusions: This study provides a preliminary in vitro dataset for comparing extracellular β-HB responses to selected dietary FAs under defined nutrient-deprived hepatocyte assay conditions. The descriptor-based analysis should be interpreted only as a small-sample, exploratory, hypothesis-generating structure–response framework.

## 1. Introduction

Ketone bodies (KBs)—including acetoacetate (AcAc), β-hydroxybutyrate (β-HB), and acetone—are produced mainly in liver mitochondria from acetyl-CoA generated largely through fatty acid (FA) β-oxidation, particularly when carbohydrate availability or insulin signaling is limited. They serve as alternative energy substrates for peripheral tissues such as the brain, heart, and skeletal muscle, and help maintain systemic energy homeostasis when glucose availability is limited [1,2]. Among them, β-HB is the most abundant circulating form. In healthy individuals, circulating KB concentrations are maintained at low levels, but they rise during prolonged fasting, exercise, or carbohydrate restriction and can become markedly elevated in pathological states such as diabetic ketoacidosis [3,4]. Therefore, circulating KB levels serve as both key metabolic biomarkers and biologically active signaling molecules.

Nutritional interventions such as the ketogenic diet (KD)—characterized by high-fat, moderate-protein, and low-carbohydrate intake—can elevate KB levels, and have been explored for the management of neurological disorders [5,6,7] and as adjuncts in cancer therapy [8,9]. Conversely, sustained pathological elevations of KB levels in poorly controlled diabetes may aggravate metabolic disturbances [10,11,12]. These dual roles underscore the need to understand how individual dietary FAs influence β-HB response, so that lipid-induced KB responses can be interpreted more cautiously within appropriate nutritional and disease contexts [13,14].

Ketogenesis occurs mainly in liver mitochondria, and FAs are major substrates for this pathway. The magnitude of extracellular β-HB response is associated with FA structure, including chain length and unsaturation pattern, because these properties can affect cellular uptake, mitochondrial entry, and β-oxidation fate [1,2,15]. However, quantitative comparisons across food-relevant FAs remain limited. Previous studies have focused on only a few representative substrates, such as medium-chain fatty acids (MCFAs) from coconut oil [16], C18:1 from olive oil, C18:2 from soybean oil, and C18:3 from flaxseed oil [17]. The β-HB responses of many distinctive food-source FAs, including odd-chain FAs from dairy fat and ruminant products [18]; very-long-chain monounsaturated fatty acids (VLCMUFAs) from some seed oils [19]; and conjugated linolenic acids from pomegranate seed oil [20] remain poorly characterized. This gap limits systematic evaluation of FA structure–associated β-HB response patterns under controlled in vitro conditions. Machine learning (ML) can help summarize molecular structure–response associations and may support hypothesis generation when applied with explicit small-sample caution [21,22,23]. Based on these gaps, it was hypothesized that hepatocellular β-HB accumulation is associated with structural features of dietary-relevant FAs, and that structure–response patterns could be summarized using an exploratory descriptor-based analysis to guide future experimental FA assessment.

In this study, an integrated in vitro assay and ML workflow was established to compare the extracellular β-HB response of dietary-relevant FAs under defined hepatocyte-derived assay conditions. Dose-dependent and time-dependent extracellular β-HB responses of 19 FAs, including 9 saturated, 4 monounsaturated, and 6 polyunsaturated FAs, were quantified in HepG2 and AML12 cells [16]. Although HepG2 and AML12 cells were used as hepatocyte-derived screening models, they do not fully reproduce primary human hepatic lipid metabolism, mitochondrial function, or whole-body physiological regulation. Therefore, the observed extracellular β-HB responses were interpreted as relative in vitro screening results rather than as direct reflections of human hepatic physiology. A GradientBoosting model was then trained using the HepG2 dataset collected at 100 μM and 12 h, and model stability was assessed using repeated random splits, leave-one-out cross-validation (LOOCV), permutation testing, MCFA sensitivity analyses, and simple baseline models based on carbon number and double-bond number. Model-dependent descriptor contribution and ranking-stability analyses were used to summarize fitted-model associations without implying biochemical causality. Finally, food-derived FAs selected from the COCONUT database through similarity matching were used only for exploratory hypothesis-list generation. This workflow summarizes chemical structure-associated extracellular β-HB responses while retaining clear boundaries between in vitro evidence, model-derived hypotheses, and future experimental validation.

## 2. Materials and Methods

### 2.1. Chemicals and Reagents

Alpha mouse liver 12 (AML12) cells, human hepatocarcinoma cells (HepG2), and their respective culture media were procured from Pricella Biotech Co., Ltd. (Wuhan, China). High-glucose DMEM media, sterile PBS, 0.25% trypsin-EDTA were acquired from Solarbio Science & Technology Co. Limited (Beijing, China). BSA (fatty acid and IgG free), and CCK-8 assay kit were acquired from Beyotime Biotechnology Co., Ltd. (Shanghai, China); Serum-, glucose-, sodium pyruvate-, and glutamine-free medium was obtained from Thermo Fisher Scientific Inc. (Cleveland, OH, USA). β-Hydroxybutyrate fluorometric assay kit was obtained from Cayman Chemical (Ann Arbor, MI, USA). C6:0 (purity ≥ 99%), C8:0 (purity ≥ 99%), C14:0 (purity ≥ 99%), C16:0 (purity ≥ 99%), C17:0 (purity ≥ 98%), and C18:1 (bio-reagent) were acquired from Sigma-Aldrich (Shanghai, China). C10:0 (purity ≥ 99.5%), C12:0 (purity ≥ 99.5%), C15:0 (analytical standard), C18:0 (purity ≥ 99.5%), C18:2 (purity ≥ 99.0%), C18:2 (9Z, 11E) (purity ≥ 98.0%), C18:2 (10E, 12Z) (purity ≥ 90.0%), C18:3 *n*-3 (purity ≥ 99.0%), C20:4 (purity ≥ 99.0%), C20:5 (EPA, analytical standard), C22:1 (purity ≥ 99.0%), C22:6 (purity ≥ 98.0%), and C24:1 (purity ≥ 99.0%) were procured from Aladdin Reagent Co., Ltd. (Shanghai, China). C16:1 (purity ≥ 98.5%) and C18:3 *n*-6 (purity ≥ 97.0%) were acquired from Shanghai McLean Biochemical Technology Co., Ltd. (Shanghai, China). C11:0 (purity ≥ 98.0%), C13:0 (purity ≥ 98.0%) were obtained from Shanghai Yuanye Biotechnology Co., Ltd. (Shanghai, China).

### 2.2. Conjugation of Fatty Acids to Bovine Serum Albumin

Given their low aqueous solubility, FAs were conjugated to albumin before cell exposure to provide a soluble delivery format commonly used in in vitro FA-treatment studies. Based on established procedures with minor modifications, a 5:1 FA/BSA molar ratio was selected because previous studies have shown that this ratio allows efficient binding of most FAs under controlled in vitro preparation conditions [24,25]. Briefly, FAs were dissolved in sodium hydroxide (FA:NaOH = 1:1.5) under continuous stirring at the following temperatures for 1.5 h: 70 °C for C15:0, C16:0, C17:0, and C18:0; 50 °C for C12:0, C14:0, C11:0, C13:0, C18:2 (9Z, 11E), C18:2 (10E, 12Z), C22:1, and C24:1; and 37 °C for C6:0, C8:0, C10:0, C16:1, C18:1, C18:2, C18:3 *n*-3, C18:3 *n*-6, C20:4, C20:5, and C22:6. This procedure yielded 2.5 mM sodium FA stock solutions. BSA was dissolved separately in PBS to obtain a 0.5 mM stock solution. The FA and BSA solutions were then mixed 1:1 (*v*/*v*), incubated at 37 °C for 1 h, adjusted to pH 7.4, filtered through a 0.22 μm membrane, and stored at −20 °C for no longer than 1 week.

The total FA concentration in the prepared FA-BSA stock solutions was verified by GC-FID after lipid extraction and derivatization. Fatty acid methyl esters were analyzed using an Agilent 7820A GC system equipped with a DB-Fast FAME column (30 m × 0.25 mm × 0.25 μm; Agilent, Santa Clara, CA, USA). This verification confirmed total FA recovery in the stock solutions but did not directly quantify the albumin-bound and unbound FA fractions or the bioavailable FA concentration delivered to cells.

### 2.3. Cellular Cultivation and Cell-Viability Assessment

HepG2 and AML12 cells were cultured in their designated media in T25 culture flasks (Corning Inc., Corning, NY, USA) at 37 °C in a humidified incubator containing 5% CO_2_ (Heracell 240i, Thermo Fisher Scientific Inc., Waltham, MA, USA). The culture medium was replaced every 2 days, and cells were passaged 2–3 times per week.

To maintain consistency with the subsequent β-HB assay conditions, cell viability was assessed under nutrient-deprived conditions using the CCK-8 method, with reference to a previous study [26]. Briefly, cells (1 × 10^6^ cells/mL) in complete medium were seeded in 96-well plates (Corning Inc., Corning, NY, USA) and incubated for 24 h. The medium was replaced with serum-, glucose-, sodium pyruvate-, and glutamine-free medium containing FA-BSA complexes at final concentrations of 0–800 μM. After incubation for 24 h (HepG2) or 8 h (AML12), 10 μL of CCK-8 reagent was added to each well, followed by incubation for 2 h, and the absorbance was measured at 450 nm using a microplate reader (SpectraMax M5, Molecular Devices, Sunnyvale, CA, USA). Cell viability was calculated according to Equation (1).

(1) $$\mathrm{Cell}\;\mathrm{viability}\;(\%)\;=\;\frac{{OD}_{sample}\;-\;{OD}_{blank}}{{OD}_{control}\;-\;{OD}_{blank}}\;\times \;100\%$$

### 2.4. Quantification of Extracellular β-HB Accumulation Induced by 19 Fatty Acids in HepG2 and AML12 Cells

Both dose-dependent and time-dependent extracellular β-HB responses were measured using a fluorometric assay kit to characterize β-HB response profiles. A blank control group containing BSA but no added FA was included in every experiment and processed using the same normalization procedure before blank subtraction. β-HB concentrations were first calculated from the standard curve. To account for differences in cell number and viability after FA exposure, β-HB values were normalized to viable-cell equivalents. Viable-cell equivalents were estimated as total cell content per well, expressed as 10^6^ cells, multiplied by relative cell viability, where relative cell viability was calculated as cell viability (%)/100. C16:0 was included on each 48-well plate as a plate-wise internal reference. The correction factor was calculated as the mean C16:0 response across plates divided by the C16:0 response on the corresponding plate. The β-HB concentration values used for endpoint calculation were normalized using this C16:0 correction factor before viable-cell correction and BSA-blank subtraction, as shown in Supplementary Table S1. The final reported values therefore represent BSA-blank-subtracted, viability-corrected, and plate-wise C16:0-normalized extracellular β-HB responses, expressed as μM/10^6^ cells. This internal-reference normalization was used to reduce plate-to-plate analytical variation, but no additional statistical batch-effect correction model was applied. Supplementary Table S1 provides the normalization workflow, C16:0-normalized β-HB values before viable-cell correction, corresponding viability and cell-content data, and the final BSA-blank-subtracted, viability-corrected β-HB endpoint values used for analysis and figure generation.

#### 2.4.1. Dose–Response Relationship of Extracellular β-HB Accumulation

This assay was performed with minor modifications to established hepatocyte β-HB response protocols [27,28,29]. HepG2 and AML12 cells were seeded into 48-well plates and incubated for 24 h. The FA concentration range was selected based on preliminary cell-viability assessment and dose–response experiments, with the aim of enabling controlled in vitro comparison among FAs while avoiding overt loss of viability under the present assay conditions. These concentrations represent nominal in vitro exposure concentrations of FA-BSA complexes and were not intended to directly mimic dietary intake or postprandial circulating FA levels. After the medium was removed, cells were washed twice with PBS and then incubated in serum-, glucose-, sodium pyruvate-, and glutamine-free medium containing FA-BSA complexes at 25, 50, 100, 200, or 400 μM. This nutrient-deprived medium was intentionally used to reduce background carbon sources and increase the dependence of cells on exogenous FA-BSA complexes, thereby improving the sensitivity of the assay for comparing FA-associated extracellular β-HB responses. However, this medium also creates a starvation-like stress condition that may affect cell viability, metabolic activity, redox state, substrate utilization, and β-HB release. Therefore, the resulting β-HB values were interpreted as apparent extracellular β-HB accumulation under defined screening conditions, not as a routine postprandial or physiological exposure response. Cells were exposed for 12 h (HepG2) or 6 h (AML12), after which β-HB in the culture medium was quantified using the fluorometric assay kit [30].

#### 2.4.2. Time-Dependent Kinetics of Extracellular β-HB Accumulation

The pretreatment procedure was identical to that described in Section 2.4.1. Based on the viability results and detectable β-HB responses from the dose–response experiments, 100 μM was selected for HepG2 cells and 200 μM for AML12 cells in subsequent time-course experiments. These cell-line-specific concentrations were selected for within-cell-line time-course comparison and were not intended for direct quantitative comparison between HepG2 and AML12 cells. After two PBS washes, cells were incubated in serum-, glucose-, sodium pyruvate-, and glutamine-free medium containing a fixed FA concentration for different durations. HepG2 cells were sampled at 4, 8, 12, 18, and 24 h, whereas AML12 cells were sampled at 1, 2, 4, 6, and 8 h. β-HB concentrations in the culture medium were then measured to characterize time-dependent β-HB response kinetics.

### 2.5. Exploratory ML Analysis of Structure-Associated β-HB Responses to FAs

ML analysis was used as a small-sample exploratory tool to examine whether molecular descriptors could support relative ranking hypotheses for FA-associated β-HB responses under the defined HepG2 assay conditions.

#### 2.5.1. Data Acquisition

The simplified molecular input line entry system (SMILES) for 19 experimentally tested FAs was obtained from the PubChem database (https://pubchem.ncbi.nlm.nih.gov/ (accessed on 13 March 2026)). The model input target was extracellular β-HB response in HepG2 cells measured at 100 μM after 12 h. This endpoint was selected because 100 μM was the highest concentration that maintained acceptable viability for all tested FAs in HepG2 cells under the present assay conditions, most FAs showed robust β-HB responses at this concentration, and the 12 h time point captured substantial β-HB accumulation while avoiding late-stage kinetic flattening for most compounds.

#### 2.5.2. Calculation of Molecular Fingerprint Similarity

Molecular fingerprints provide a way to characterize chemical structures and are commonly employed in molecular similarity assessments and clustering analyses. The open-source cheminformatics software RDKit (version 2026.03.2, http://www.rdkit.org/ (accessed on 23 March 2026)) was used to generate ECFP4 fingerprints from the SMILES strings of each FA. Hierarchical clustering of FAs was performed using the single-link algorithm of the Python scikit-learn library (version 1.7.2, https://scikit-learn.org.cn/ (accessed on 27 March 2026)), and the Tanimoto coefficient-based similarity analysis was used to compute pairwise similarity among all substances [31].

#### 2.5.3. Calculation of Molecular Descriptors and Processing of Features

**Figure 1:**
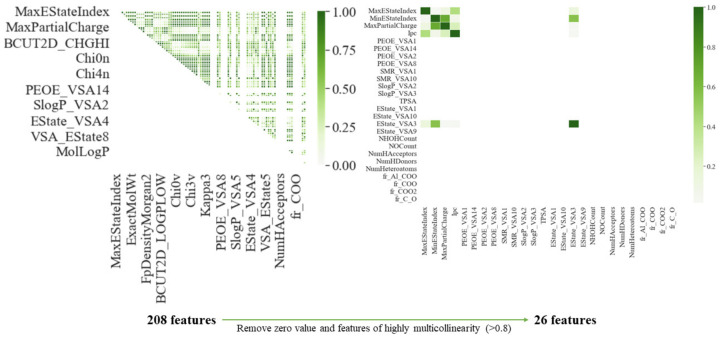
Descriptive molecular descriptor filtering workflow. RDKit descriptors were calculated from FA SMILES strings. Descriptors with zero variance and descriptors showing high multicollinearity (|r| > 0.8) were removed, yielding 26 descriptors for the descriptive feature-processing summary.

Because of the limited sample size, directly using high-dimensional fingerprints as model inputs would increase the risk of overfitting. Therefore, RDKit molecular descriptors were used as candidate predictors instead of high-dimensional fingerprints [32]. A total of 208 descriptors were calculated for all FA molecules with the MolecularDescriptorCalculator module in RDKit. For the descriptive feature-processing summary shown in Figure 1, descriptors with zero variance and descriptors showing high multicollinearity (|r| > 0.8) were removed, yielding a final set of 26 descriptors for model development. For all model-selection and assessment procedures, however, all-zero descriptor removal and correlation filtering were refitted within the relevant training partition or cross-validation fold to reduce information leakage. No missing β-HB values or missing descriptor values were present in the model-development dataset.

#### 2.5.4. Regression Modeling and Model Assessment

The resulting descriptor matrix was converted into a DataFrame, and regression models for extracellular β-HB responses were constructed using the scikit-learn library. The complete 19-FA dataset was randomly divided into a training set and a four-compound held-out subset at an 80:20 ratio. The training set was used for model development and model selection, whereas the held-out subset was excluded from model fitting and retained only for descriptive held-out evaluation. The exact training/held-out subset assignment is reported in Supplementary Table S2.

Model selection was performed within the training set using 5-fold cross-validation. Twelve regression algorithms were initially evaluated and reported in Supplementary Table S9. In the leakage-controlled 5-fold cross-validation procedure, all-zero descriptor removal and correlation filtering were refitted within each training fold before validation. Under this conservative assessment, the candidate models showed unstable cross-validation results; therefore, no algorithm was used to claim robust predictive superiority. GradientBoostingRegressor was retained as the final exploratory model because it can capture potential non-linear descriptor–response relationships, supports model-dependent descriptor-contribution analysis, and provided continuity with the original model framework. Accordingly, it was used to summarize fitted descriptor–response associations rather than as a validated optimal predictor. The final GradientBoostingRegressor was refitted using the complete 15-compound training set, and its single-split predictions and residuals are reported in Supplementary Table S3. Candidate-model screening results and final model settings are provided in Supplementary Table S9.

To assess split-dependent instability, 1000 repeated random 80:20 splits were performed, with descriptor filtering, model training, and held-out evaluation repeated within each training/held-out partition. Assessment-result distributions were summarized using the median, interquartile range, and 2.5–97.5 percentile intervals (Supplementary Table S4). LOOCV was performed as an internal stability assessment (Supplementary Table S5), and a 1000-iteration permutation test was conducted by randomly permuting β-HB values and recalculating LOOCV Q^2^ (Supplementary Table S6).

To assess whether the descriptor-based model mainly captured simple structural properties, baseline models using only carbon number and number of double bonds were fitted, including LinearRegression, Ridge regression, Lasso regression, and PLSRegression. Their LOOCV results were compared with those of the descriptor-based GradientBoosting model (Supplementary Table S7). MCFA-excluded and MCFA-withheld sensitivity analyses were further performed to evaluate the dependence of model assessment results on the MCFA/LCFA contrast (Supplementary Table S8).

Model assessment was based on R^2^, MAE, MSE, and RMSE (Equations (2)–(5)) [33,34]. For LOOCV, the cross-validated coefficient of determination was reported as Q^2^. Prediction-error behavior was summarized using residual tables, and descriptive calibration-style summaries (Supplementary Tables S3 and S5). These summaries were used only for transparent error reporting and were not interpreted as formal clinical calibration or evidence of clinical utility. Because of the 19-compound sample size, the 26-descriptor feature space, and the lack of a diverse external chemical assessment set, the ML analysis was interpreted only as an exploratory, hypothesis-generating framework for relative ranking under defined in vitro conditions.

(2) $$R^{2}=1-\frac{{SS}_{res}}{{SS}_{tot}}=1-\frac{{{\sum_{i}(y_{i}-f}_{i})}^{2}}{{\sum_{i}(y_{i}-\bar{y})}^{2}}$$

(3) $$MAE=\frac{1}{n}\sum_{i=1}^{n}\left|(y_{i}-f_{i})\right|$$

(4) $$MSE=\frac{1}{n}\sum_{i=1}^{n}{\left(y_{i}-f_{i}\right)}^{2}$$

(5) $$RMSE=\sqrt{\frac{1}{n}\sum_{i=1}^{n}{\left(y_{i}-f_{i}\right)}^{2}}$$

In these equations, y*_i_* denotes the actual value and f*_i_* signifies the predicted value, respectively. SS*_res_* represents the sum of squared residuals, while SS*_tot_* signifies the total sum of squares. *n* represents the sample size.

#### 2.5.5. Assessment of Model-Dependent Descriptor Contribution and Ranking Stability 

Descriptor contribution was examined for the refitted GradientBoostingRegressor to summarize model-dependent associations between molecular descriptors and fitted β-HB outputs. Descriptor-ranking stability was further assessed across repeated random splits by calculating the frequency with which each descriptor appeared among the five highest-ranked contributors. These analyses were interpreted only as exploratory fitted-model associations and were not used to infer biochemical causality or mechanistic determinants of extracellular β-HB accumulation.

#### 2.5.6. COCONUT-Based Exploratory FA Ranking and Preliminary Assessment of Four Additional FAs

The MolNatSim tool based on the COCONUT natural product database was used to identify natural FAs structurally similar to the original 19-FA experimental set [34]. Compounds with molecular similarity greater than 80% were retained and manually screened for food-source relevance. The food-derived FAs were then scored with the refitted GradientBoosting to generate an exploratory ranked hypothesis list within a chemically similar domain.

Four commercially available FAs that were not included among the 19 model-development FAs, namely C11:0, C13:0, C18:2 (9Z, 11E), and C18:2 (10E, 12Z), were further tested in HepG2 cells as a preliminary experimental ranking assessment under the same assay conditions. These compounds were not used for descriptor filtering, algorithm selection, hyperparameter tuning, descriptor interpretation, or refitting of the GradientBoosting Regressor. Owing to their limited number, structural similarity to the original FA set, and testing within the same laboratory system, this analysis was not considered an independent external model-assessment experiment. Instead, it was used only to compare the experimentally observed relative responses with the exploratory model-derived ranking within a chemically similar domain.

### 2.6. Data Processing and Analysis

Data are presented as mean ± SD from three independent biological experiments. Statistical comparisons were performed using one-way analysis of variance (ANOVA) followed by Tukey-adjusted multiple comparisons. For dose–response experiments, comparisons were conducted within each cell line and concentration, or within each fitted dose–response parameter. For time-course experiments, comparisons were conducted within each cell line and time point. Data from different cell lines, doses, and time points were not pooled into a single global ANOVA; therefore, significance labels should be interpreted as within-cell-line and within-condition comparisons. Statistical significance was defined as *p* < 0.05. Dose–response and time-course figures were generated using R software (version 4.5.1; R Foundation for Statistical Computing, Vienna, Austria) with the ggplot2 package. Shaded areas in the line plots represent 95% confidence intervals calculated from the three independent biological experiments. Heatmaps were constructed using TBtools-II (version II).

## 3. Results and Discussion

### 3.1. Selection of Working Exposure Concentrations in HepG2 and AML12 Cells

Working exposure ranges with acceptable viability (>80%) were first established to ensure that subsequent β-HB measurements primarily reflected intrinsic metabolic responses rather than overt cellular stress.

**Figure 2:**
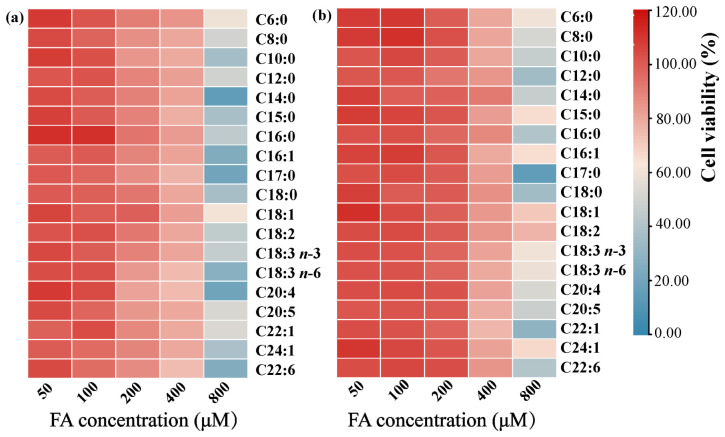
Viability assessment and selection of working concentrations for β-hydroxybutyrate assays. Heatmaps showing the viability of HepG2 cells (**a**) and AML12 cells (**b**) after treatment with FA-BSA complexes at 50–800 μM. HepG2 cells were exposed for 24 h, whereas AML12 cells were exposed for 8 h. Cell viability was measured using the CCK-8 assay and expressed as a percentage of the untreated control group, which was set to 100%. The color scale indicates relative cell viability (%), with lower values indicating greater reduction in viability.

As shown in Figure 2, HepG2 and AML12 cells were treated with FAs at concentrations ranging from 0 to 800 μM. Under the present assay conditions, viability remained approximately 80% at 400 μM in both cell lines, indicating measurable viability reduction at higher exposure levels. Based on these observations, 100 μM for HepG2 cells and 200 μM for AML12 cells were selected as working concentrations that balanced measurable β-HB responses with acceptable cell viability.

Importantly, these concentrations are nominal in vitro comparison values and do not represent physiological circulating FA levels. Circulating free FA concentrations in humans are commonly reported to range from 0.1 to 0.6 mmol/L [35] and daily supplementation with 20 mL MCT oil has been reported to raise circulating MCFA concentrations above 100 µM in healthy humans [36]. These reports were used only to contextualize the nominal exposure range and do not imply that the present single-FA, nutrient-deprived cell assay reproduces dietary or postprandial FA exposure.

### 3.2. Dose–Response Relationship of Extracellular β-HB Accumulation for 19 FAs in HepG2 and AML12 Cells

#### 3.2.1. Dose-Dependent β-HB Response Profiles

**Figure 3:**
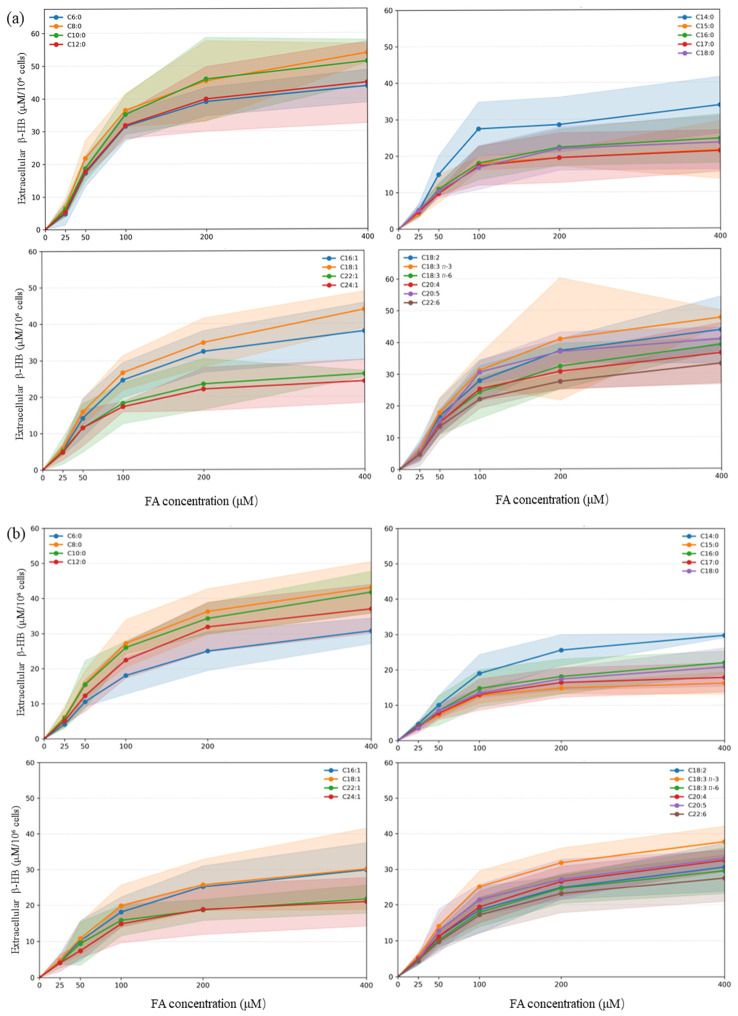
Dose-dependent extracellular β-hydroxybutyrate (β-HB) accumulation induced by fatty acids in HepG2 (**a**) and AML12 cells (**b**). HepG2 cells were treated with FA-BSA complexes at 25–400 μM for 12 h, whereas AML12 cells were treated with FA-BSA complexes at 25–400 μM for 6 h. β-HB values were BSA-blank-subtracted, viability-corrected, C16:0-normalized, and normalized to 10^6^ cells. Different colored lines and symbols represent individual FAs as indicated in the legends, and the shaded bands in the corresponding colors represent the 95% confidence intervals. Data are presented as the means of three independent experiments.

Figure 3 displays the concentration-dependent β-HB response curves for 19 FAs in HepG2 (Figure 3a) and AML12 (Figure 3b) cells, and Table 1 summarizes key parameters including maximal extracellular β-HB response (peak of the sigmoidal curve), and half-maximal effective concentration (EC50) values. Across both cell lines, a consistent structure-associated response pattern was observed: MCFAs exhibited the highest apparent extracellular β-HB responses, several 18-carbon unsaturated FAs showed comparatively high responses, and long-chain saturated FAs (LCSFAs) and very long-chain monounsaturated FAs (VLCMUFAs) were the lowest (*p* < 0.05), with other MUFAs and PUFAs demonstrating intermediate levels.

In HepG2 cells, most FAs exhibited sigmoidal dose–response curves characteristic of concentration-dependent β-HB response. C8:0 and C10:0 produced the highest maximal β-HB responses and exceeded the response of C16:0 by more than two-fold (*p* < 0.05), with EC50 values of approximately 68–70 μM. Polyunsaturated FAs (PUFAs) demonstrated moderate-to-high β-HB responses. C18:3 *n*-3 showed the highest maximal response (approximately 1.92-fold vs. C16:0), followed closely by C18:1 (approximately 1.89-fold vs. C16:0) and C18:2 (approximately 1.82-fold vs. C16:0). However, very long-chain PUFAs (e.g., C20:4, C22:6) showed significantly lower peak β-HB response (34.85–37.34 μM; *p* < 0.05). By contrast, LCSFAs such as C15:0, C17:0, C18:0, VLCMUFAs such as C22:1, and C24:1 showed much lower maximal responses; C14:0 also produced significantly more β-HB than C16:0, indicating that modest shortening of the saturated chain was associated with a higher β-HB response in this assay.

A similar ranking was observed in AML12 cells. C8:0 and C10:0 again produced the highest maximal β-HB responses (approximately 47 μM; 1.99-fold and 1.95-fold vs. C16:0, respectively; EC50 = 83.10 μM and 83.73 μM, respectively; *p* < 0.05), followed by C18:3 *n*-3 (40.55 μM; approximately 1.71-fold vs. C16:0; EC50 = 77.00 μM; *p* < 0.05). In contrast, LCSFAs such as C15:0, C17:0 and VLCMUFAs such as C22:1, C24:1 showed relatively low responses. Thus, the overall structure-dependent pattern was preserved across the two hepatocyte-derived cell models.

The maximal β-HB response reflects the upper level of extracellular β-HB accumulation reached under the assay conditions, whereas the EC50 value describes the concentration required to achieve half-maximal stimulation. Extracellular β-HB accumulation generally increased steeply between 25 and 100 μM, rose more slowly between 100 and 200 μM, and approached a plateau near 400 μM. In HepG2 cells, maximal responses ranged from 21.37 to 55.54 μM and EC50 values between 48.93 and 89.87 μM. In AML12 cells, maximal responses ranged from 16.83 to 47.21 μM and EC50 values ranged from 56.76 to 96.84 μM.

These observations indicate that relative β-HB response rankings were broadly consistent between HepG2 and AML12 cells, supporting an association between FA structure and extracellular β-HB responses under the present in vitro assay conditions. At the same time, differences in absolute response magnitude likely reflect cell-line-specific variation in FA uptake, oxidative metabolism, and overall metabolic phenotype.

Direct comparison of absolute β-HB levels between HepG2 and AML12 cells should remain cautious. HepG2 cells are human hepatocarcinoma-derived cells and may differ from primary human hepatocytes in differentiation status, mitochondrial function, and lipid metabolic capacity. AML12 cells are non-tumorigenic murine hepatocyte-derived cells that retain several hepatic features, but their murine origin introduces species-specific differences in fatty acid uptake, β-oxidation, mitochondrial substrate handling, and ketone-body metabolism may affect direct comparison with HepG2 cells [16]. In addition, the two models were tested at different working concentrations and sampling durations. Therefore, cross-cell-line consistency was interpreted mainly as support for relative FA ranking under defined in vitro conditions, whereas absolute β-HB levels were considered cell-line- and protocol-dependent. Accordingly, the observed extracellular β-HB responses should be interpreted as relative response patterns under defined hepatocyte-derived in vitro screening conditions, providing a controlled basis for hypothesis generation rather than a direct substitute for primary human hepatic physiology.

#### 3.2.2. Structure-Associated β-HB Response Patterns

The dose–response results showed that extracellular β-HB responses were associated with FA chain length and unsaturation under the present in vitro assay conditions. The main observed pattern was that C8:0 and C10:0 produced the highest responses, several 18-carbon unsaturated FAs showed comparatively high responses, and several LCSFAs and VLCMUFAs showed lower responses.

A possible explanation for the high responses of C8:0 and C10:0 is their relatively rapid entry into hepatic oxidative pathways. Previous studies have demonstrated pronounced β-HB responses to MCFAs [16,37], and these FAs are less dependent than long-chain FAs on the carnitine shuttle for mitochondrial utilization. By contrast, C6:0 yields fewer acetyl-CoA units per molecule, whereas C12:0 may behave metabolically more like a long-chain substrate. These properties may partly explain why both C6:0 and C12:0 showed lower β-HB responses than C8:0 and C10:0 (*p* < 0.05).

The lower β-HB responses of saturated LCFAs may be explained by structural features that influence mitochondrial utilization. Saturated LCFAs are more dependent on CPT1-mediated mitochondrial entry and may undergo slower oxidative utilization. C18:0, in particular, has been reported to show lower CPT1 affinity, which may contribute to its limited β-HB response in the present assay [38,39]. Odd-chain LCFAs (C15:0, C17:0) generate propionyl-CoA during β-oxidation, which can be converted into succinyl-CoA entering the TCA cycle. This route may reduce the relative contribution of these substrates to acetyl-CoA availability for β-HB formation. In addition, malonyl-CoA may indirectly regulate hepatic β-HB response by inhibiting CPT1-mediated mitochondrial entry and oxidation of long-chain acyl-CoAs [40]. For VLCMUFAs, chain-shortening through peroxisomal β-oxidation before efficient mitochondrial oxidation may introduce additional metabolic steps compared with shorter-chain substrates, potentially reducing their contribution to extracellular β-HB accumulation. These explanations remain inferential because FA uptake, acyl-CoA formation, CPT1 activity, β-oxidation flux, oxygen consumption, and acetyl-CoA flux were not directly measured.

For PUFAs, the observed responses were not explained by unsaturation alone. Several PUFAs showed higher β-HB responses than LCSFAs, which may be partly related to previously reported differences in oxidation rates [41,42] and peroxisome proliferator-activated receptor alpha (PPARα)-associated lipid-oxidation pathways [43,44,45]. However, PPARα activation and ketogenic-enzyme expression were not measured in this study. Among PUFAs, C18:2 and C18:3 *n*-3 showed comparatively high β-HB responses, consistent with literature reporting relatively efficient oxidation [46]. In contrast, very-long-chain PUFAs (VLCPUFAs) may require coordinated peroxisomal and mitochondrial β-oxidation, which could contribute to their lower responses under the present assay conditions. The relatively low β-HB response of C20:4 may be related to its reported slower peroxisomal oxidation and preferential incorporation into phospholipids [47]. Similarly, limited hepatic oxidative availability of C22:6 may reduce acetyl-CoA precursor supply and extracellular β-HB accumulation [48].

Taken together, these observed data indicate that FA-induced β-HB responses were related to an integrated structural balance rather than to chain length or unsaturation alone. Under the present assay conditions, MCFAs served as reference substrates for rapid β-HB accumulation, whereas the relatively high response of C18:3 *n*-3 [49] and the lower responses of several LCSFAs and VLCMUFAs provide hypotheses for follow-up biochemical testing. These interpretations require further validation using additional ketone-body endpoints, metabolic-flux assays, digestion models, animal studies, and human studies.

### 3.3. Time-Dependent Response Relationship of β-HB Accumulation

**Figure 4:**
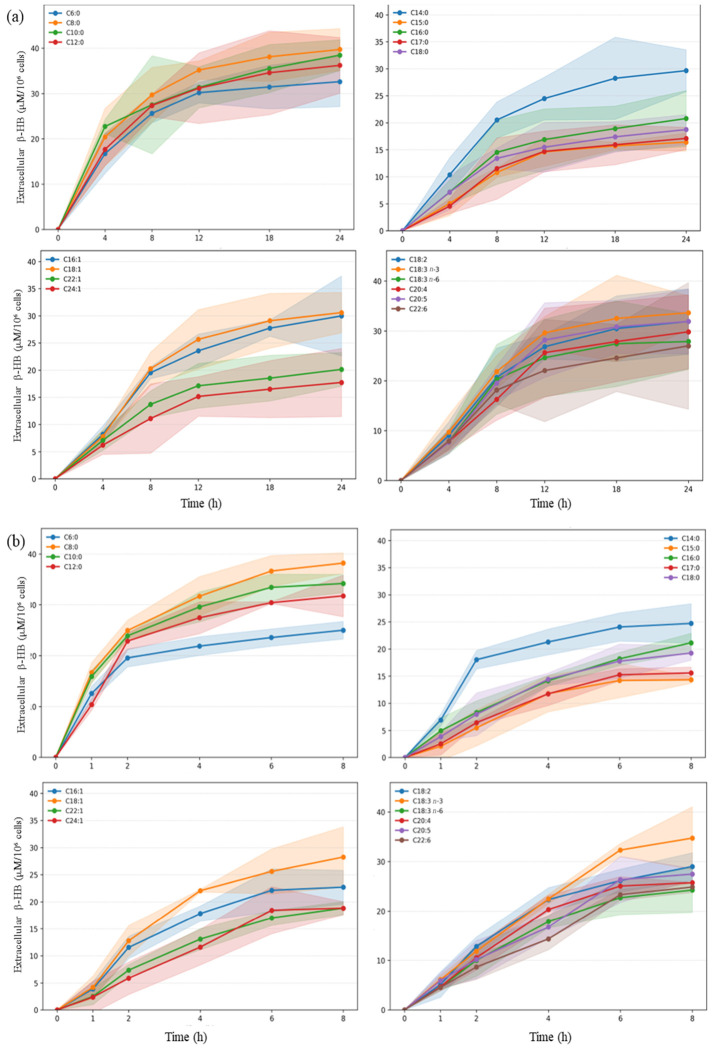
Time-dependent accumulation of β-hydroxybutyrate (β-HB) induced by 19 fatty acids in HepG2 cells at 100 μM (**a**) and AML12 cells at 200 μM (**b**). β-HB values were BSA-blank-subtracted, viability-corrected, C16:0-normalized, and normalized to 10^6^ cells. HepG2 cells were treated with 100 μM FA-BSA complexes and sampled at 0, 4, 8, 12, 18, and 24 h, whereas AML12 cells were treated with 200 μM FA-BSA complexes and sampled at 0, 1, 2, 4, 6, and 8 h. Different colored lines and symbols represent individual FAs as indicated in the legends, and the shaded bands in the corresponding colors represent the 95% confidence intervals based on three independent experiments.

Dose–response analysis provides a static estimate of extracellular β-HB accumulation, whereas time-course analysis describes the appearance of β-HB in the medium after FA exposure. Figure 4 therefore extends the experimental comparison from response magnitude to apparent response kinetics. The time-course analysis provided useful within-system information on apparent extracellular β-HB accumulation kinetics. However, because HepG2 and AML12 cells do not fully reproduce primary human hepatic lipid metabolism, mitochondrial β-oxidation, ketogenic-enzyme activity, or integrated metabolic regulation, these results should be interpreted as response patterns under defined in vitro screening conditions.

In HepG2 cells (Figure 4a), MCFAs, especially C8:0 and C10:0, maintained the highest β-HB levels at all measured time points, and their apparent accumulation rates peaked at 4 h. Although β-HB concentrations continued to rise up to 24 h, the rate of increase progressively declined thereafter. C14:0 showed a similar early kinetic pattern, also reaching its maximal apparent accumulation rate at 4 h. Most other LCSFAs reached their peak apparent accumulation rates at 8 h, after which their rates declined. Most MUFAs and PUFAs displayed similar kinetics: a gradual increase from 0 to 4 h, a faster increase from 4 to 8 h and a slower increase thereafter. Very long-chain PUFAs such as C20:4 displayed one of the most delayed responses, with peak accumulation occurring at 12 h.

In AML12 cells (Figure 4b), the temporal separation between fast- and slow-responding substrates was even more evident. MCFAs triggered a rapid initial response, with peak accumulation rates occurring at 1 h for C6:0, C8:0, and C10:0 and at 2 h for C12:0. C8:0 and C10:0 showed the highest initial accumulation rate, with subsequent increases occurring at markedly reduced rates. C14:0 behaved more similarly to the MCFAs than to the longer saturated FAs, peaking at 2 h. By contrast, C16:0 and C18:0 reached their highest accumulation rates at 4 h, and C15:0 and C17:0 showed both slow accumulation and low overall β-HB levels (2.50 ± 0.83 μM at 1 h to 15.58 ± 0.44 μM at 8 h). Among the MUFAs, C18:1 showed the highest rate, whereas C22:1 and C24:1 remained low. PUFAs showed kinetics similar to those of MUFAs, with rapid increases from 0 to 1 h, increased accumulation between 1 and 2 h, and peak rates at 4 h. At this time point, C18:3 *n*-3 exhibited the highest rate, whereas C20:4 had the lowest.

Overall, time-course results showed that β-HB accumulation varied dynamically over time under the present nutrient-deprived assay conditions. Most FAs induced a time-dependent increase in β-HB levels that either stabilized or fluctuated mildly afterward. The observed temporal ranking was broadly consistent with the dose–response results, with MCFAs showing faster early responses and several LCFAs showing slower accumulation patterns.

For interpretation, the faster response of MCFAs may be related to their lower dependence on CPT-mediated mitochondrial entry, whereas LCFAs may require additional uptake, activation, and mitochondrial entry processes before contributing to extracellular β-HB accumulation. Over longer durations, β-HB accumulation rates may stabilize or decline because of cellular adaptation, substrate depletion, altered redox state, or stress-related metabolic inhibition. Therefore, these time-course results mainly provide within-system information on FA-associated extracellular β-HB accumulation patterns and may guide follow-up studies of rapid or sustained β-HB responses to selected FAs.

### 3.4. Exploratory ML Modeling of FA-Associated β-HB Structure–Response Relationships

The SMILES notation, representative natural sources, and extracellular β-HB responses for the 19 FAs used in the modeling analysis are summarized in Table 2. For the modeling analysis, the experimentally generated β-HB dataset was used to summarize structure–response patterns and generate relative ranking hypotheses. The modeling endpoint was supported by the observation that many FAs showed broadly similar relative β-HB response trends across the two cell models. The HepG2 12 h dataset at 100 μM was selected as the modeling endpoint because this condition produced robust β-HB responses while maintaining acceptable viability and captured substantial accumulation before late-stage kinetic slowing. For interpretation, AML12 data were used only to examine cross-cell-line consistency of the overall ranking, whereas the ML model was trained using a single standardized HepG2 endpoint to avoid cell-line- and protocol-dependent confounding.

#### 3.4.1. Exploratory Model Assessment

The exact training/held-out subset assignment is provided in Supplementary Table S2. In the single 80:20 split, the held-out subset contained only four FAs. This split yielded an apparent held-out R^2^ of 0.875, and single-split estimates and residuals are reported in Supplementary Table S3, with descriptive estimation-error summaries provided in Supplementary Table S11. For interpretation, this value was considered statistically fragile because it depended on the identity of only four withheld compounds. Therefore, the single-split result was retained only as a descriptive sensitivity check and not as evidence of robust generalization.

**Figure 5:**
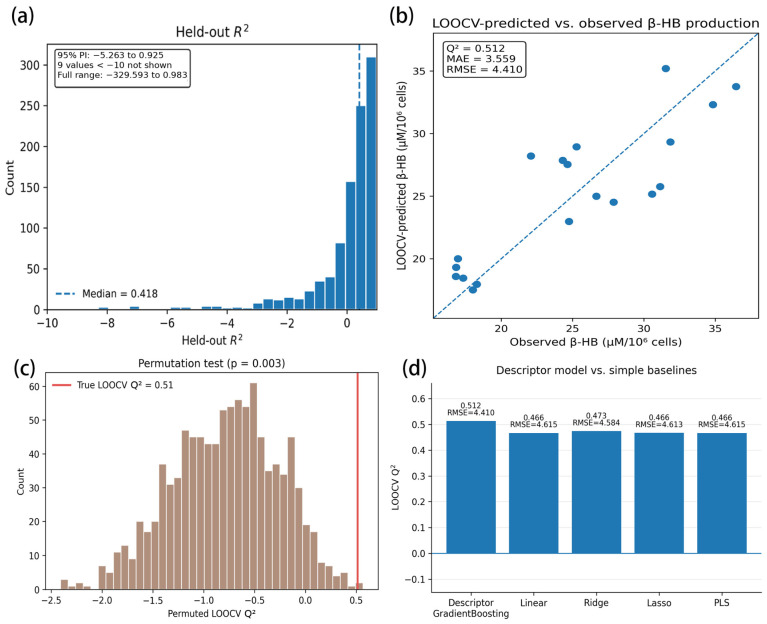
Exploratory assessment of model robustness and overfitting risk. (**a**) Distribution of held-out R^2^ values from 1000 repeated random 80:20 splits. The wide distribution indicates that apparent held-out assessment results were highly dependent on which fatty acids were assigned to the four-compound held-out subset. (**b**) LOOCV-estimated vs. observed extracellular β-HB responses for the descriptor-based GradientBoosting model. Each blue circle represents an individual FA. (**c**) Permutation test comparing the true LOOCV Q^2^ with the null distribution generated by 1000 random permutations of the β-HB response values. (**d**) LOOCV assessment comparison between the descriptor-based GradientBoosting model and simple two-variable baseline models using only carbon number and number of double bonds. β-HB, β-hydroxybutyrate; LOOCV, leave-one-out cross-validation; Q^2^, cross-validated coefficient of determination.

The repeated-split analysis provided a clearer assessment of partition-dependent variability. Across 1000 repeated random 80:20 splits, held-out assessment results varied substantially, with a median held-out R^2^ of 0.418 and a 2.5–97.5 percentile interval from −5.263 to 0.925 (Figure 5a; Supplementary Table S4). The repeated-split distributions for MAE and RMSE further showed that estimation error varied across data partitions. These observations indicate that apparent held-out assessment results were highly dependent on the four compounds assigned to the held-out subset.

LOOCV provided a complementary internal estimate of small-sample model stability. The descriptor-based GradientBoosting model yielded LOOCV Q^2^ = 0.512, MAE = 3.559, and RMSE = 4.410 (Figure 5b; Supplementary Table S5). A 1000-iteration permutation test showed that the observed LOOCV Q^2^ was higher than the permutation distribution (median permuted Q^2^ = −0.752; 2.5–97.5 percentile interval = −1.840 to 0.122; permutation *p* = 0.003; Figure 5c; Supplementary Table S6). This result suggests that the model captured some descriptor–response structure rather than purely random associations, although it does not establish robust external generalizability.

The baseline-model comparison showed that simple two-variable models using only carbon number and number of double bonds performed similarly to the descriptor-based GradientBoosting model. For example, the Ridge baseline achieved LOOCV Q^2^ = 0.473 and RMSE = 4.584, close to the descriptor-based GradientBoosting model (Q^2^ = 0.512; RMSE = 4.410; Figure 5d; Supplementary Table S7). LinearRegression, Lasso regression, and PLSRegression showed comparable assessment results. These observations indicate that chain length and unsaturation explained a substantial part of the observed β-HB response pattern and that the descriptor-based GradientBoosting model did not demonstrate robust predictive superiority over simple structural baseline models in this small dataset. Thus, the ML workflow mainly summarized structure-associated trends that were partly expected from basic FA structural features, rather than providing evidence of a validated predictive model.

The MCFA-excluded and MCFA-withheld sensitivity analyses further evaluated dependence on the MCFA/LCFA contrast (Supplementary Table S8). In the MCFA-excluded analysis, the model was refitted and evaluated after removing the MCFA subset, thereby reducing the structural contrast available to the model. In the more stringent MCFA-withheld analysis, the model was trained only on non-MCFAs and then used to estimate responses for withheld MCFAs (C6:0, C8:0, C10:0, and C12:0); assessment results decreased markedly (R^2^ = −15.108; RMSE = 8.285), indicating unreliable extrapolation to an under-represented chemical class. Therefore, these sensitivity analyses support the use of the descriptor model only as an exploratory relative-ranking tool within the represented chemical space, not as a robust quantitative predictor. Compound-level uncertainty intervals were not considered reliable given the very small dataset; future larger datasets should report uncertainty intervals for individual scored compounds.

Calibration-style and residual analyses were reported only for descriptive estimation-error assessment. Descriptive slope/intercept summaries, residual statistics, LOOCV estimated-versus-observed plots, residual tables, and repeated-split assessment distributions are provided in Figure 5 and Supplementary Tables S3–S5 and S11. These analyses were not interpreted as formal clinical calibration and were not used to claim clinical utility or deployment readiness because the present model is a molecular descriptor-based exploratory ranking model rather than a clinical or diet-level assessment model.

**Table 2:** SMILES notation, representative natural sources, and extracellular β-hydroxybutyrate responses in HepG2 cells after treatment with 19 dietary fatty acids at 100 μM.

	Fatty Acids	SMILES	Natural Sources	β-HB (μM/10^6^ Cells)
1	C6:0	CCCCCC(=O)O	dairy fat	31.53
2	C8:0	CCCCCCCC(=O)O	coconut oil	36.46
3	C10:0	CCCCCCCCCC(=O)O	coconut oil	34.84
4	C12:0	CCCCCCCCCCCC(=O)O	coconut oil	31.85
5	C14:0	CCCCCCCCCCCCCC(=O)O	dairy fat	24.75
6	C15:0	CCCCCCCCCCCCCCC(=O)O	dairy fat	16.97
7	C16:0	CCCCCCCCCCCCCCCC(=O)O	palm oil	18.01
8	C16:1	CCCCCC/C=C\CCCCCCCC(=O)O	fish oil	24.64
9	C17:0	CCCCCCCCCCCCCCCCC(=O)O	dairy fat	17.33
10	C18:0	CCCCCCCCCCCCCCCCCC(=O)O	animal fats	16.82
11	C18:1	CCCCCCCC/C=C\CCCCCCCC(=O)O	olive oil	26.67
12	C18:2	CCCCC/C=C\C/C=C\CCCCCCCC(=O)O	soybean oil	27.88
13	C18:3 *n*-3	CC/C=C\C/C=C\C/C=C\CCCCCCCC(=O)O	flaxseed oil	31.14
14	C18:3 *n*-6	CCCCC/C=C\C/C=C\C/C=C\CCCCC(=O)O	evening primrose oil	24.31
15	C20:4	CCCCC/C=C\C/C=C\C/C=C\C/C=C\CCCC(=O)O	animal fats	25.28
16	C20:5	CC/C=C\C/C=C\C/C=C\C/C=C\C/C=C\CCCC(=O)O	fish oil	30.57
17	C22:1	CCCCCCCC/C=C\CCCCCCCCCCCC(=O)O	rapeseed oil	18.29
18	C22:6	CC/C=C\C/C=C\C/C=C\C/C=C\C/C=C\C/C=C\CCC(=O)O	fish oil	22.08
19	C24:1	CCCCCCCC/C=C\CCCCCCCCCCCCCC(=O)O	acer truncatum seed	16.84

Values represent the mean extracellular β-hydroxybutyrate responses from three independent biological replicates, each measured in triplicate.

#### 3.4.2. Model-Dependent Descriptor Contribution and Ranking Stability Analysis

**Figure 6:**
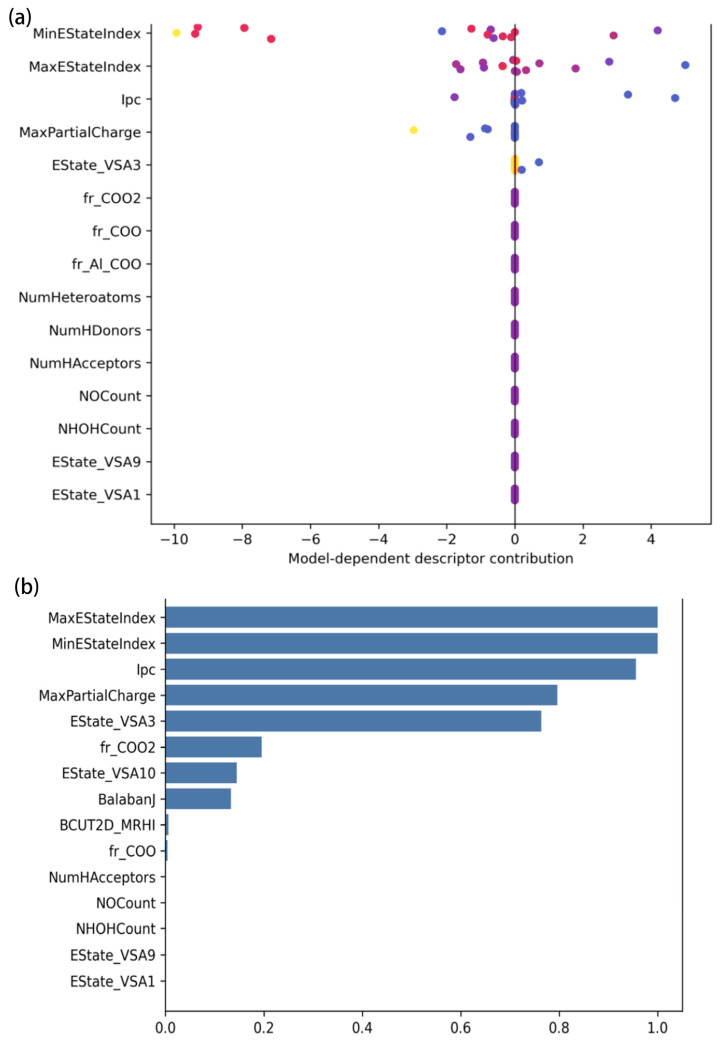
Model-dependent descriptor contribution and ranking stability analysis. (**a**) Model-dependent descriptor contribution summary for the refitted GradientBoosting model. Each point represents the model-dependent contribution for an individual FA, and the point colors indicate the relative descriptor values within each descriptor, with darker purple–blue colors representing lower values, red–pink colors representing intermediate values, and yellow colors representing higher values. (**b**) Descriptor ranking stability across repeated random splits, expressed as the frequency with which each descriptor appeared among the top five contributors. MaxEStateIndex, MinEStateIndex, Ipc, MaxPartialCharge, and EState_VSA3 were most frequently ranked among the top contributors. These results were interpreted as exploratory, model-dependent associations in a small dataset rather than as biochemical mechanisms or causal determinants of extracellular β-HB responses.

The descriptor contribution analysis identified the descriptors most strongly associated with fitted model outputs under the refitted GradientBoosting model. The highest-ranked descriptors included MinEStateIndex, MaxEStateIndex, Ipc, MaxPartialCharge, and EState_VSA3 (Figure 6a).

The ranking-stability analysis showed that MinEStateIndex, MaxEStateIndex, Ipc, MaxPartialCharge, and EState_VSA3 appeared most frequently among the top five contributors across repeated random splits (Figure 6b; Supplementary Table S10). This observation suggests that the same broad descriptor families recurred across resampling runs. Nevertheless, because only 19 FAs were available, these rankings remain model-dependent and sample-dependent.

#### 3.4.3. Exploratory COCONUT-Derived FA Ranking and Preliminary Experimental Assessment

The COCONUT-based workflow was used only for exploratory hypothesis-list generation within a chemically similar and food-relevant FA domain. Food-derived FAs with >80% structural similarity to the experimental set were retrieved and manually filtered for food-source relevance. Table 3 summarizes the model-derived 12 h extracellular β-HB ranking estimates in HepG2 cells for 18 food-derived FAs selected through this workflow [50]. This list included both unmeasured candidate FAs and four additional FAs that were experimentally tested. As a descriptive observation from this ranking list, MCFAs, such as C9:0 and C12:1, tended to show higher model-derived β-HB scores, whereas very-long-chain saturated FAs, including C20:0, C22:0, and C24:0, remained low. Several long-chain unsaturated FAs were placed in an intermediate range. These values should be regarded as in silico ranking scores within the represented chemical space, not as quantitative predictions, the discovery of bioactive lipid ingredients, evidence of nutritional efficacy, or a basis for practical lipid selection.

Four commercially available FAs from this exploratory list, namely C11:0, C13:0, C18:2 (9Z, 11E), and C18:2 (10E, 12Z), were further tested in HepG2 cells to provide limited experimental context for the model-derived ranking estimates. Their measured extracellular β-HB responses and model-derived ranking estimates are shown in Table 3. Among these four compounds, the measured responses followed the same general relative order as the model-derived ranking estimates, with C11:0 showing the highest response and C18:2 (10E, 12Z) showing the lowest response. However, because only four structurally related compounds were tested and all measurements were performed in the same laboratory assay system, this comparison should be interpreted only as a preliminary experimental assessment within a chemically similar FA domain.

Overall, Table 3 should be read as an exploratory hypothesis list supported by limited experimental context, not as evidence of independent external model assessment or a demonstration of quantitative prediction. The model-derived values for the unmeasured FAs are in silico ranking scores within the represented chemical space and should not be interpreted as validated β-HB values, evidence of nutritional efficacy, discovery of bioactive lipid ingredients, or a basis for practical lipid selection. Larger and more chemically diverse experimental datasets will be required before model generalizability, quantitative prediction or applied lipid-selection claims can be considered.

### 3.5. Limitations and Future Directions

The present study provides preliminary in vitro evidence that selected dietary FAs differ in their extracellular β-HB responses under defined nutrient-deprived hepatocyte assay conditions. Chain length and unsaturation pattern appeared to be associated with relative β-HB responses in this system. However, the descriptor-based analysis should be interpreted only as an exploratory, hypothesis-generating summary of structure–response patterns, not as a validated model of dietary ketogenic potential, clinical relevance, or applied nutritional efficacy.

The present single-FA FA-BSA exposure model does not reproduce actual dietary lipid intake. In foods, FAs are consumed mainly as components of complex lipid mixtures and are largely esterified in triacylglycerols within food matrices. Therefore, the present findings should be interpreted as apparent extracellular β-HB responses under standardized in vitro conditions, not as direct evidence for dietary ketogenic effects, functional lipid design, precision nutrition applications, or clinical practice. Any potential relevance to dietary lipid effects, functional lipid design, or precision nutrition should therefore be considered only as a future perspective requiring validation in more physiologically relevant cell models, digestion and food-matrix systems, animal models, and human dietary studies.

Several experimental limitations should be acknowledged. First, HepG2 cells are widely used in hepatic metabolism studies, but they are hepatocarcinoma-derived and do not fully reproduce the differentiation state, lipid metabolic capacity, mitochondrial function, or ketogenic activity of primary human hepatocytes. AML12 cells provide a non-tumorigenic hepatocyte-derived comparison model, but they are of murine origin and may differ from human hepatocytes in FA uptake, β-oxidation, mitochondrial substrate handling, and KB metabolism. Therefore, the observed β-HB responses should be interpreted as relative FA-associated response patterns under defined hepatocyte-derived in vitro screening conditions, providing preliminary information for further validation in more physiologically relevant models. Second, extracellular β-HB accumulation was the only ketone-body endpoint measured. Acetoacetate, total KBs, β-HB/acetoacetate ratio, β-oxidation flux, CPT1 activity, HMGCS2, HMGCL, and BDH1, PPARα activation, oxygen consumption, and isotope-tracing endpoints were not assessed. Therefore, the present results cannot define the complete activation state or mechanistic regulation of the hepatic ketogenic pathway. Third, although β-HB values were corrected for relative viability and normalized to 10^6^ cells, this correction may influence the apparent magnitude of β-HB responses when cell viability decreases. Therefore, Supplementary Table S1 provides β-HB values before viable-cell correction, corresponding viability and cell-content data, and final corrected endpoint values. Nevertheless, this correction does not fully account for FA-induced changes in metabolic activity, mitochondrial function, redox balance, ATP status, or cellular stress. Fourth, the FA-BSA delivery system represents another limitation. Although GC-FID confirmed total FA recovery in the prepared FA-BSA stock solutions, albumin-bound and unbound FA fractions were not quantified. Because individual FAs may differ in albumin binding, solubility, cellular uptake, and intracellular availability, the nominal FA-BSA concentration may not fully reflect the actual bioavailable FA concentration delivered to cells. Thus, differences in apparent extracellular β-HB responses may reflect both intrinsic metabolic handling and FA-specific delivery or availability under the present assay conditions.

The ML analysis also remains statistically limited. The model was developed using only 19 FAs, retained an unfavorable descriptor-to-sample ratio, and lacked a diverse external chemical assessment set or independent replication. Repeated random-split assessment, LOOCV, permutation testing, MCFA sensitivity analyses, simple baseline comparisons, and prediction-error summaries improved reporting transparency but cannot overcome the small-sample limitation. Moreover, the similar results of simple baseline models using only carbon number and double-bond number indicated that the descriptor-based GradientBoosting model did not demonstrate robust predictive superiority. Therefore, the ML workflow should be interpreted mainly as an exploratory summary of structure-associated trends partly expected from chain length and unsaturation, rather than as a validated predictive model. Future studies should expand the FA dataset, include structurally diverse compounds, test mixed-lipid and structured-triacylglycerol systems, incorporate digestion and food-matrix models, measure additional metabolic endpoints, and evaluate more physiologically relevant cell models, animal models, and human dietary interventions before any nutritional or translational relevance can be established.

## 4. Conclusions

In conclusion, this study established a preliminary hepatocyte-derived in vitro screening approach for comparing apparent extracellular β-HB accumulation induced by selected dietary FAs under defined nutrient-deprived conditions. C8:0 and C10:0 induced the highest apparent responses in both HepG2 and AML12 cells, whereas several long-chain saturated FAs and very-long-chain MUFAs showed lower responses, providing a controlled dataset for exploring FA structure-associated β-HB response patterns. Because only extracellular β-HB was measured and HepG2/AML12 cells do not fully reproduce primary human hepatic lipid and mitochondrial metabolism, these findings should be interpreted as relative within-system screening results rather than as evidence of complete ketogenic-pathway activation or human hepatic ketone-body physiology. The descriptor-based modeling summarized the observed patterns, but repeated random-split assessment, LOOCV, permutation testing, MCFA sensitivity analyses, and simple baseline comparisons showed unstable assessment results and no robust predictive superiority over simple structural features such as carbon number and unsaturation. Therefore, the model should be regarded only as a small-sample, hypothesis-generating tool, not as evidence for dietary ketogenic effects, in vivo responses, or clinical/nutritional outcomes. Future studies should expand the FA panel, incorporate additional mechanistic endpoints, and validate findings in more physiologically relevant systems—including primary human hepatocytes, digestion and food matrix models, animal studies, and human dietary interventions—before any nutritional or translational implications can be drawn.

## Figures and Tables

**Table 1:** Maximum response values, EC50 values and R^2^ values of fitted fatty acid concentration-β-hydroxybutyrate response curves in HepG2 cells and AML12 cells.

	Fatty Acids	HepG2				AML12	
		Maximum Response Values (μM/10^6^ Cells)	EC50 (μM)	R^2^	Maximum Response Values (μM/10^6^ Cells)	EC50 (μM)	R^2^
1	C6:0	44.20	63.38	0.9939	35.20	96.84	0.9907
2	C8:0	55.54	68.09	0.9877	47.21	83.10	0.9892
3	C10:0	53.04	69.89	0.9903	46.30	83.73	0.9901
4	C12:0	45.68	64.62	0.9858	40.69	84.65	0.9864
5	C14:0	32.63	53.37	0.9729	32.68	83.11	0.9908
6	C15:0	21.38	48.93	0.9776	16.83	56.76	0.9894
7	C16:0	25.66	59.08	0.9831	23.70	73.54	0.9775
8	C17:0	21.37	51.03	0.9728	18.90	62.41	0.9806
9	C18:0	25.08	62.61	0.9763	22.72	78.45	0.9860
10	C16:1	40.31	75.23	0.9885	33.81	93.29	0.9824
11	C18:1	48.58	89.87	0.9890	32.35	75.72	0.9714
12	C22:1	27.64	63.74	0.9790	22.88	62.07	0.9788
13	C24:1	25.36	59.68	0.9813	22.27	70.26	0.9663
14	C18:2	46.66	76.43	0.9824	34.04	84.22	0.9812
15	C18:3 *n*-3	49.25	71.72	0.9750	40.55	77.00	0.9930
16	C18:3 *n*-6	42.41	82.36	0.9873	32.90	89.46	0.9892
17	C20:4	37.34	67.55	0.9841	36.99	95.78	0.9936
18	C20:5	41.08	63.41	0.9942	34.49	74.95	0.9831
19	C22:6	34.85	72.17	0.9876	30.36	85.46	0.9855

**Table 3:** Preliminary ranking-check data and COCONUT-derived exploratory hypothesis list in HepG2 cells at 100 μM for 12 h.

	Fatty Acids	SMILES	Natural Sources	Model-Derived β-HB Ranking (μM/10^6^ Cells)	Observed β-HB Response (μM/10^6^ Cells)
1	C9:0	CCCCCCCCC(=O)O	dairy fat	34.43	-
2	C11:0	CCCCCCCCCCC(=O)O	dairy fat	32.44	29.65 ± 1.31
3	C12:1	CCCCCC/C=C\CCCC(=O)O	coconut oil	32.49	-
4	C13:0	CCCCCCCCCCCCC(=O)O	animal fat	27.83	25.77 ± 0.67
5	C14:1	CCCC/C=C\CCCCCCCC(=O)O	dairy fat	25.24	-
6	C15:1	CCCC/C=C\CCCCCCCCC(=O)O	animal fat	25.25	-
7	C18:2 (9, 11)	CCCCCC\C=C\C=C/CCCCCCCC(=O)O	animal fat	29.53	26.89 ± 0.82
8	C18:2 (10, 12)	CCCCC/C=C/C=C/CCCCCCCCC(=O)O	animal fat	26.59	24.62 ± 0.54
9	C18:3 (9, 11, 13)	CCCC\C=C/C=C/C=C\CCCCCCCC(=O)O	punica granatum seed oil	29.32	-
10	C18:3 (8, 10, 12)	CCCCC/C=C/C=C/C=C/CCCCCCC(=O)O	calendula officinalis seed oil	29.38	-
11	C20:0	CCCCCCCCCCCCCCCCCCCC(=O)O	peanut oil	16.83	-
12	C20:1	CCCCCCCC/C=C\CCCCCCCCCC(=O)O	rapeseed oil	26.44	-
13	C20:2	CCCCC/C=C\C/C=C\CCCCCCCCCC(=O)O	rapeseed oil	26.65	-
14	C20:3 (5, 11, 14)	CCCCC/C=C\C/C=C\CCCC/C=C\CCCC(=O)O	pine nut oil	25.73	-
15	C22:0	CCCCCCCCCCCCCCCCCCCCCC(=O)O	peanut oil	16.84	-
16	C22:2 (13, 16)	CCCCC/C=C\C/C=C\CCCCCCCCCCCC(=O)O	marine fish	25.91	-
17	C22:3 (13, 16, 19)	CCC=CCC=CCC=CCCCCCCCCCCCC(=O)O	marine fish	26.23	-
18	C24:0	CCCCCCCCCCCCCCCCCCCCCCCC(=O)O	peanut oil	16.84	-

Note: “-” indicates not experimentally measured. Measured values are presented as mean ± SD from three independent biological replicates.

## Data Availability

The processed β-HB endpoint values, cell-viability data, processed descriptor list and model-input summary, training/held-out assignment, model predictions and residuals, repeated-split and LOOCV outputs, prediction-error summaries, final model settings, and code/data availability information are provided or summarized in Supplementary Tables S1–S11. The Python scripts used for descriptor calculation, model fitting, repeated-split validation, LOOCV, permutation testing, baseline modeling, and sensitivity analyses, as well as the R scripts used for dose–response and time-course figure generation, are available from the corresponding author upon reasonable request. The code and descriptor-processing information are provided for transparency and reproducibility of this exploratory analysis and do not constitute a deployable prediction tool.

## Supplementary Materials

The following supporting information can be downloaded at: https://www.mdpi.com/article/10.3390/nu18122021/s1. Supplementary Table S1. β-HB normalization workflow, non-viability-corrected β-HB values, cell-viability data, cell-content values, and final processed β-HB endpoint values. Supplementary Table S2. Training/held-out subset assignment for the 19-fatty-acid HepG2 model-development dataset. Supplementary Table S3. Single 80:20 split predictions and residuals for the 19-fatty-acid HepG2 model-development dataset. Supplementary Table S4. Performance results from 1000 repeated random splits. Supplementary Table S5. LOOCV compound-level predictions and residuals for the 19-fatty-acid HepG2 model-development dataset. Supplementary Table S6. Permutation-test results for LOOCV Q^2^. Supplementary Table S7. Comparison between descriptor-based GradientBoosting and simple structural baseline models. Supplementary Table S8. MCFA-excluded and MCFA-withheld sensitivity analyses for the 19-fatty-acid HepG2 model-development dataset. Supplementary Table S9. Candidate model screening and final GradientBoosting model settings. Supplementary Table S10. Model-dependent descriptor contribution and descriptor-ranking stability. Supplementary Table S11. Descriptive prediction-error and residual-diagnostic summaries.

## Abbreviations

The following abbreviations are used in this manuscript:

AcAc	Acetoacetate
CoA	Coenzyme A
COCONUT	COCONUT natural product database
CPT1	Carnitine palmitoyltransferase 1
ECFP4	Extended-connectivity fingerprint 4
FA	Fatty acid
FA-BSA	Fatty acid–bovine serum albumin complex
FFA	Free fatty acid
HMGCS2	3-Hydroxy-3-methylglutaryl-CoA synthase 2
Ipc	Information Content Index
KB	Ketone body
LCFA	Long-chain fatty acid
LCSFA	Long-chain saturated fatty acid
MCFA	Medium-chain fatty acid
MCT	Medium-chain triglyceride
ML	Machine learning
MUFA	Monounsaturated fatty acid
PUFA	Polyunsaturated fatty acid
QSAR	Quantitative structure–activity relationship
VLCMUFA	Very-long-chain monounsaturated fatty acid
VLCPUFA	Very-long-chain polyunsaturated fatty acid
β-HB	β-Hydroxybutyrate
