# Supplementary material for: Preliminary In Vitro Screening of Structure-Dependent β-Hydroxybutyrate Responses to Dietary Fatty Acids in Hepatocyte Models
Source: Nutrients. 2026 Jun 21;18(12):2021. doi: 10.3390/nu18122021 (PMC13305192; doi:10.3390/nu18122021)
Supplement: Supplementary file 1 [file nutrients-18-02021-s001.zip › Supplementary Table S2 and supplementary Table S3.pdf]

Supplementary Table S2. Training/held-out subset assignment for the 19-fatty-acid HepG2 model-development dataset

Compound ID	Fatty acid	Data subset
1	C6:0	held-out subset
2	C8:0	held-out subset
3	C10:0	training set
4	C12:0	training set
5	C14:0	training set
6	C15:0	held-out subset
7	C16:0	training set
8	C16:1	training set
9	C17:0	training set
10	C18:0	training set
11	C18:1	training set
12	C18:2	held-out subset
13	C18:3 n-3	training set
14	C18:3 n-6	training set
15	C20:4	training set
16	C20:5	training set
17	C22:1	training set
18	C22:6	training set
19	C24:1	training set

Note: Compound IDs correspond to those listed in Table 2. The held-out subset comprised four fatty acids from the single 80:20 split and was used only for descriptive sensitivity analysis, rather than as robust external validation.

Supplementary Table S3. Single 80:20 split predictions and residuals for the 19-fatty-acid HepG2 model-development dataset.

Data subset	Compound ID	Fatty_acid	SMILES	Observed β-HB	Predicted β-HB	Residual
held-out subset	1	C6:0	CCCCCC(=O)O	31.53	35.72485	-4.19485
held-out subset	2	C8:0	CCCCCCCC(=O)O	36.46	34.43258	2.027418
training set	3	C10:0	CCCCCCCCCC(=O)O	34.84	34.82859	0.011412
training set	4	C12:0	CCCCCCCCCCCC(=O)O	31.85	31.84396	0.006039
training set	5	C14:0	CCCCCCCCCCCCCC(=O)O	24.75	24.74745	0.002547
held-out subset	6	C15:0	CCCCCCCCCCCCCCC(=O)O	16.97	18.1098	-1.1398
training set	7	C16:0	CCCCCCCCCCCCCCCC(=O)O	18.01	18.01273	-0.00273
training set	8	C16:1	CCCCCC/C=C\CCCCCCCC(=O)O	24.64	24.66375	-0.02375
training set	9	C17:0	CCCCCCCCCCCCCCCCC(=O)O	17.33	17.32944	0.000565
training set	10	C18:0	CCCCCCCCCCCCCCCCCC(=O)O	16.82	16.83254	-0.01254
training set	11	C18:1	CCCCCCCC/C=C\CCCCCCCC(=O)O	26.67	26.65165	0.018354
held-out subset	12	C18:2	CCCCC/C=C\C/C=C\CCCCCCCC(=O)O	27.88	29.53458	-1.65458
training set	13	C18:3 n-3	CC/C=C\C/C=C\C/C=C\CCCCCCCC(=O)O	31.14	31.1119	0.028095
training set	14	C18:3 n-6	CCCCC/C=C\C/C=C\C/C=C\CCCCC(=O)O	24.31	24.31719	-0.00719
training set	15	C20:4	CCCCC/C=C\C/C=C\C/C=C\C/C=C\CCCC(=O)O	25.28	25.3076	-0.0276
training set	16	C20:5	CC/C=C\C/C=C\C/C=C\C/C=C\C/C=C\CCCC(=O)O	30.57	30.54587	0.024134
training set	17	C22:1	CCCCCCCC/C=C\CCCCCCCCCCCC(=O)O	18.29	18.29052	-0.00052
training set	18	C22:6	CC/C=C\C/C=C\C/C=C\C/C=C\C/C=C\C/C=C\CCC(=O)O	22.08	22.08948	-0.00948
training set	19	C24:1	CCCCCCCC/C=C\CCCCCCCCCCCCCC(=O)O	16.84	16.84734	-0.00734

Note: Observed β-HB values represent BSA-blank-subtracted, viability-corrected, and plate-wise C16:0-normalized extracellular β-HB responses measured in HepG2 cells at 100 μM after 12 h. The held-out subset contained four fatty acids from a single 80:20 split and was used only for descriptive held-out evaluation, not as robust external validation. Residuals were calculated as observed minus predicted β-HB values
